# Microwave Ablation Versus Radiofrequency Ablation for Treatment of Hepatocellular Carcinoma: A Meta-Analysis of Randomized Controlled Trials

**DOI:** 10.3390/cancers12123796

**Published:** 2020-12-16

**Authors:** Antonio Facciorusso, Mohamed A. Abd El Aziz, Nicola Tartaglia, Daryl Ramai, Babu P. Mohan, Christian Cotsoglou, Sara Pusceddu, Luca Giacomelli, Antonio Ambrosi, Rodolfo Sacco

**Affiliations:** 1 Department of Medical Sciences, Gastroenterology Unit, University of Foggia, 71122 Foggia, Italy; antonio.facciorusso@virgilio.it; 2 Department of Surgery, Mayo Clinic, Rochester, MN 55902, USA; abdelmaksoud.mohamed@mayo.edu; 3 Department of Medical Sciences, General Surgery Unit, University of Foggia, 71122 Foggia, Italy; nicola.tartaglia@unifg.it (N.T.); antonio.ambrosi@unifg.it (A.A.); 4 Gastroenterology and Hepatology, Brooklyn Hospital Medical Center, Brooklyn, NY 11201, USA; dramai@tbh.org; 5 Gastroenterology & Hepatology, University of Utah Health, Salt Lake City, UT 84132, USA; babu.pappumohan@hsc.utah.edu; 6 General Surgery Department, ASST-Vimercate, 20871 Vimercate, Italy; christian.cotsoglou@asst-vimercate.it; 7 Fondazione IRCCS—Istituto Nazionale dei Tumori Via G. Venezian 1 IT, 20133 Milan, Italy; sara.pusceddu@istitutotumori.mi.it; 8 Department of Surgical Sciences and Integrated Diagnostics, University of Genoa, 16126 Genoa, Italy; luca.giacomelli@polistudium.it; 9 Polistudium SRL, 20124 Milan, Italy

**Keywords:** HCC, RFA, MWA, survival, recurrence, liver cancer

## Abstract

**Simple Summary:**

Through a meta-analysis of seven randomized-controlled trials we found difference in terms of complete response and survival rates between microwave ablation (MWA) and radiofrequency ablation (RFA). While local recurrence rate was similar between MWA and RFA, distant recurrence rate was significantly lower with MWA. As a consequence, disease-free survival at 1, 2, and 3 years was similar between the two groups whereas disease-free survival at 5 years was significantly in favor of MWA. Adverse event rate was similar between the two treatments. Our results indicate a similar efficacy and safety profile between the two techniques. MWA seems to decrease the rate of long-term recurrences, but this finding needs to be confirmed in further trials.

**Abstract:**

There are limited and discordant results on the comparison between microwave ablation (MWA) and radiofrequency ablation (RFA) for the treatment of hepatocellular carcinoma (HCC). This meta-analysis aims to compare the two treatments in terms of efficacy and safety, based on a meta-analysis of randomized-controlled trials (RCTs). A computerized bibliographic search was performed on the main databases throughout August 2020. The primary outcome was the complete response rate, while survival rate (at 1-, 2-, 3-, and 5-year), disease-free survival rate (at 1-, 2-, 3-, and 5-year), local and distant recurrence rate, adverse event rate, and number of treatment sessions were the secondary outcomes. Seven RCTs enrolling 921 patients were included. No difference in terms of complete response between the two treatments was observed (risk ratio (RR) 1.01, 95% CI 0.99–1.02). Survival rates were constantly similar, with RRs ranging from 1.05 (0.96–1.15) at 1 year to 0.91 (0.81–1.03) at 5 years. While local recurrence rate was similar between MWA and RFA (RR 0.70, 0.43–1.14), distant recurrence rate was significantly lower with MWA (RR 0.60, 0.39–0.92). Disease-free survival at 1, 2, and 3 years was similar between the two groups with RR 1.00 (0.96–1.04), 0.94 (0.84–1.06), and 1.06 (0.93–1.21), respectively. On the other hand, RR for disease-free survival at 5 years was significantly in favor of MWA (3.66, 1.32–42.27). Adverse event rate was similar between the two treatments (RR 1.06, 0.48–2.34), with bleeding and hematoma representing the most frequent complications. Our results indicate a similar efficacy and safety profile between the two techniques. MWA seems to decrease the rate of long-term recurrences, but this finding needs to be confirmed in further trials.

## 1. Introduction

Hepatocellular carcinoma (HCC) is the fifth most commonly occurring type of cancer, and is the leading cause of mortality in cirrhotic patients [1].

Although up to 60% of HCC patients in developed countries are currently amenable to curative therapies, such as surgical or ablative treatments, at the time of diagnosis [2,3], tumor recurrence and long-term survival remain unsolved issues [4].

In the last few years, imaging-guided ablative therapies have gained a fundamental role in the treatment of HCC, due to their safety and efficacy, leading to complete necrosis of the tumoral nodule. Among them, percutaneous radiofrequency ablation (RFA) has become the standard-of-care for unresectable early HCCs and has been even found to be competitive with surgery in the case of a single nodule less than 3 cm in size [5,6]. However, the significant incidence of local and distant recurrences was found to affect survival [7,8] and, although several prognostic predictors of post-treatment outcomes have been extensively studied [9], other competitive ablation therapies were tested and introduced into clinical practice.

Among the more recent ablative techniques, microwave ablation (MWA) has gained a pivotal role as a valuable alternative to RFA for thermal ablation of HCC [10]. The main advantages of MWA technology, compared with other thermal ablation technologies, include consistently higher intratumoral temperatures, larger tumor ablation volumes, faster ablation times, and an improved convection profile, and, as a consequence, lower risk of a heat-sink effect (i.e., the treatment outcome is less affected by vessels in proximity to the tumor) [3].

Optimizing the treatment effects of percutaneous ablative therapies is of paramount importance for patient survival, particularly given the lack of effective adjuvant agents to use in order to decrease the risk of tumor recurrence. In fact, after the failure of the multicenter STORM randomized-controlled trial (RCT) [11], several other pharmacological agents have been tested with discordant results [4,12], hence definitive recommendations on the use of adjuvant regimens after radical therapies for HCC cannot be drawn.

After a preliminary meta-analysis published by our group claiming the non-superiority of MWA over RFA [13], several other systematic reviews and meta-analyses have been published in the last few years with discordant results [14,15,16,17]. However, these meta-analyses were based mainly on retrospective or non-randomized series; given the recent publication of several RCTs in the field, we decided to perform an updated meta-analysis restricted only to RCTs in order to provide robust and definitive evidence on the comparison between MWA and RFA.

## 2. Results

### 2.1. Literature Search and Characteristics of Included Studies

**Figure 1:**
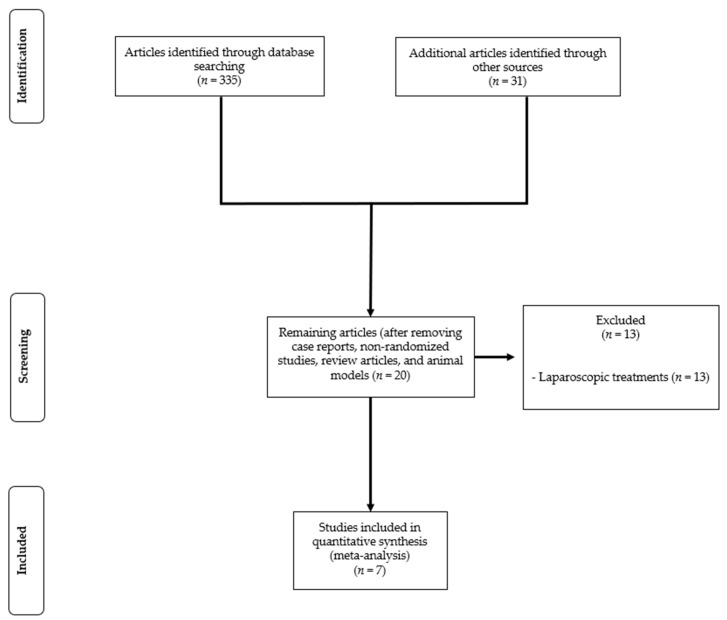
Flow chart of the search strategy.

Figure 1 shows the flow chart of the search strategy conducted in this meta-analysis.

Out of 366 studies initially identified, after exclusion of literature reviews, case reports, non-randomized studies, and animal models, 20 potentially relevant studies were extracted. After exclusion of 13 trials testing laparoscopic or surgical ablative techniques, seven RCTs were finally included in the meta-analysis [18,19,20,21,22,23,24].

Table 1 and Table S1 show the main characteristics of the included studies.

The recruitment period ranged from 1999 to 2018. Four trials [19,21,22,24] were conducted in Asia, two in Egypt [18,20], and one in Europe [23]. Overall, 473 patients were treated with MWA and 448 with RFA. The proportion of patients with multifocal or bilobar neoplasia ranged from 0% in the trial by Qian et al. [21] to 35% in the European trial [23].

Overall, the two arms were well-balanced in terms of clinical and tumoral parameters in the included trials. The mean max nodule size ranged from 1.8 to 3.28 cm, and the mean age of recruited patients was between 52 and 68 years. The majority of treated patients were male and with performance status 0. The number of patients in Child–Pugh stages A and B was equally distributed in the two treatment arms across the included trials and only a single patient in Child–Pugh stage C was recruited in the trial by Chong et al. [19].

The etiology of underlying chronic liver disease was predominantly viral and mean alpha-fetoprotein levels ranged between 20 and 282.4 ng/dL.

### 2.2. Quality Assessment

Quality assessment of the included trials is depicted in Figure S1A,B. All the studies were rated at high risk of performance bias due to unblinding of participants and personnel; however, 3 RCTs [19,22,23] were deemed as high-quality studies, whereas the quality of 4 trials [18,20,21,24] was downrated as low quality due to selection and detection bias.

### 2.3. Complete Response

Tumor response was evaluated in all of the included studies [18,19,20,21,22,23,24]. The pooled complete ablation rate was 96.1% (93.2–98.9%) with MWA and 97.2% (95.4–99%) with RFA.

**Figure 2:**
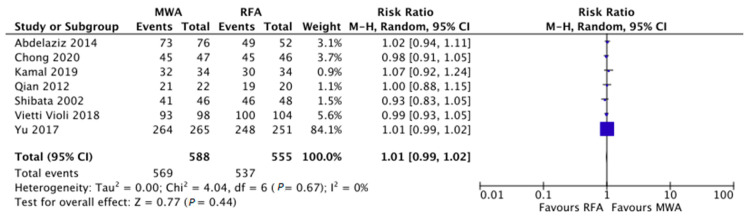
Risk ratio concerning the comparison between microwave and radiofrequency ablation in terms of complete response was 1.01 (95% confidence interval 0.99–1.02), with no evidence of heterogeneity (I^2^ = 0%).

As reported in Figure 2, RR concerning the comparison between MWA and RFA in terms of complete response was 1.01 (95% CI 0.99–1.02), with no evidence of heterogeneity (I^2^ = 0%). No significant publication bias was found by means of visual examination of the funnel plot.

In order to further confirm these findings, a sensitivity analysis was performed with three different subgroups analyses, as reported in Table 2. The comparability between the two treatments was confirmed in all of the subsets tested, based on nodule size (≤3 cm versus >3 cm), study location (East versus West/Africa), and restricted to high-quality trials. Heterogeneity was mainly low or moderate in all of the comparisons (Table 2). In particular, pooled complete ablation rate in tumors ≤3 cm was 97.9% (95.3–100%) with MWA and 97.4% (94.4–100%) with RFA; on the other hand, pooled rates of complete ablation in tumors >3 cm were 80.1% (59.7–100%) and 80.7% (57–100%) with the two treatments, respectively.

Meta-regression confirmed the lack of correlation between the proportion of patients with multifocal neoplasia (Figure S2A) and mean nodule size (Figure S2B) and the RRs for complete response.

### 2.4. Survival Outcomes

Data on survival rate and the other secondary outcomes are reported in Table 3.

Survival rates at all of the tested time-points were comparable between the two treatments. In particular, RR at 1 year was 1.05 (95% CI 0.96–1.15), at 2 years it was 1.03 (0.89–1.18), at 3 years it was 0.99 (0.91–1.09), and at 5 years it was 0.91 (0.81–1.03). Heterogeneity was mainly low (Table 3).

No evidence of publication bias was found.

The number of treatment sessions needed to obtain complete response was similar between the two groups (mean difference 0.08, 95% CI −0.07 to 0.23), although this finding should be interpreted with caution due to the high heterogeneity observed (I^2^ = 92%; Table 3).

### 2.5. Recurrence and Safety

While local recurrence rate was similar between MWA and RFA, with an RR of 0.70 (0.43–1.14), distant recurrence rate was significantly lower with MWA (RR 0.60, 0.39–0.92; I^2^ = 0%) based on three RCTs [18,20,21].

Disease-free survival at 1, 2, and 3 years was similar between the two groups with RR 1.00 (0.96–1.04), 0.94 (0.84–1.06), and 1.06 (0.93–1.21), respectively. On the other hand, RR for disease-free survival at 5 years was significantly in favor of MWA (3.66, 1.32–42.27; Table 3).

Again, no significant publication bias concerning this outcome was found.

Pooled rates of complications were 3.3% (1.7–4.9%) and 2.8% (1.3–4.3%) with MWA and RFA, respectively. The adverse event rate was similar between the two treatments, with an observed RR of 1.06 (0.48–2.34; I^2^ = 13%).

The detailed list of adverse events registered in the included trials is reported in Table 4. The most frequent complications were bleeding and hematoma. Of note, no adverse event was observed in the trial by Qian et al. [21].

## 3. Discussion

Locoregional treatments represent the first-line treatment option in patients with early HCC, thus meaning a single nodule <5 cm or up to 3 nodules <3 cm, not suitable for surgical therapy.

The first ablative technique used was percutaneous ethanol injection (PEI), able to induce coagulative necrosis of the target lesion. Although PEI was proved to be as effective as RFA in nodules <2 cm, the higher recurrence rate has limited its use in nodules located in “at-risk” segments, such as adjacent to the abdominal wall or other organs [3]. Subsequently, thermal ablative therapies emerged, including RFA, MWA, and laser ablation. Most procedures are performed using a percutaneous approach, which allows for performing these techniques even in poor surgical candidates.

Several series and meta-analyses have compared the two techniques with discordant results. A previous meta-analysis published by our group showed similar efficacy between the two therapies, with a significant benefit of MWA only in larger neoplasms [13].

Other more recent meta-analyses found a significant benefit in terms of reduced local recurrence rates with MWA, although whether MWA significantly prolongs overall survival in HCC patients remained unclear [25,26].

Given the recent publication of several RCTs comparing the two techniques, we decided to address this unsolved issue through a meta-analysis restricted to seven RCTs in order to provide definitive data able to inform the guidelines.

We think a meta-analysis of only RCTs might help to address this important question as it enables us to overcome several selection and performance biases due to the retrospective nature of most of the studies included in previous systematic reviews.

We found several key observations. First, MWA and RFA determined a similar rate of complete tumor ablation (RR 1.01), a finding confirmed even in the subgroup of larger neoplasms (>3 cm). This aspect represents a novel result, thus demonstrating that RCTs failed to confirm previous observations, based mainly on retrospective series, of an expected superiority of MWA in ablating larger tumors due to the larger area of necrosis that can be achieved with MWA [13,25,26]. Furthermore, only a slight non-significant difference in terms of the number of sessions needed to obtain complete response was observed, and hence RFA was found to provide similar results as compared to MWA in an equal number of sessions.

Second, survival rates at different time point up to 5 years after treatment were similar between the two groups, which is similar to the current literature [13,25,26]. Third, while local recurrence rate was similar between MWA and RFA, distant recurrences were observed significantly less frequently with MWA (RR 0.60, 0.39–0.92). As a consequence, even if short-term disease-free survival was similar between the two groups, disease-free survival at 5 years was significantly in favor of MWA (RR 3.66, 1.32–42.27). This finding represents a further element of novelty in our analysis, suggesting that MWA is more effective in decreasing the risk of de novo malignancy, which usually occurs beyond 2 years from the treatment and in different liver segments. In fact, local recurrences are mainly due to true relapses of the previously treated nodule, and the similar local recurrence rate observed in our meta-analysis seems to suggest a comparable ablation efficacy on the target nodule between the two techniques. On the other hand, the lower distant recurrence rate observed with MWA might be related to the wider area of necrosis achieved with this therapy, which is more likely to induce necrosis of microsatellites, well-known to be able to lead to de novo distant relapses. However, these findings should be interpreted with caution due to the high heterogeneity and because it is based on only 2 RCTs [19,24]. Further RCTs reporting long-term outcomes are urgently needed to confirm this observation.

Fourth, the adverse event rate was low and similar between the two treatments, thus confirming that both the ablative techniques are safe with a very low incidence of major complications. Of note, the vast majority of recruited patients were in Child–Pugh stage A or B, which represents the limit within a curative therapy that can be offered to an HCC patient.

There are some limitations to our study. First, the number of trials and recruited patients was relatively low and some subgroup analyses, for example according to Child–Pugh or BCLC stage, could not be performed due to the lack of available data. However, we decided to restrict our analysis only to RCTs in order to provide definitive and robust data, not biased by the retrospective nature of the included studies. Second, the quality of the included studies was mainly moderate or low, mainly due to performance bias. However, this bias is not avoidable in trials testing a new technique, as the operator cannot be blinded to the technique that he/she is actually using. Furthermore, it should be noted that objective outcomes such as survival or tumor recurrence are highly unlikely to be biased by the lack of blinding of the operator. Analysis of some long-term outcomes was based on a limited number of trials (usually 2 studies) and this might represent a further limitation and a potential source of heterogeneity.

Third, the analysis of the costs was beyond the scope of the manuscript, therefore we cannot make definitive assumptions in this regard.

Despite these limitations, our study has a number of strengths. It represents the first meta-analysis of RCTs comparing the two main percutaneous thermal ablation techniques for liver primary tumors. Moreover, the current study provides a comprehensive and simultaneous assessment of therapeutic efficacy, in terms of complete response, local/distant recurrence rate and overall survival, and of the safety profile of the two treatments. Third, any possible source of heterogeneity that could have influenced the final results was explored by means of appropriate statistical tools and the main findings were confirmed performing sensitivity analysis and meta-regression.

## 4. Materials and Methods

### 4.1. Search Strategy and Selection Criteria

This systematic review is reported according to the Preferred Reporting Items for Systematic Reviews and Meta-Analyses (PRISMA) statement and was conducted following a priori established protocol [27].

Studies included in this meta-analysis were RCTs that met the following inclusion criteria: (a) patients: adults HCC patients treated with (b) interventions: percutaneous MWA or (c) comparator: percutaneous RFA and reported (d) outcome: complete ablation of the treated nodules, defined as complete response (CR).

We excluded (a) observational or non-randomized studies, and (b) RCTs comparing laparoscopic or open surgical ablative treatments.

The search strategy was conducted in main databases throughout August 2020, based on the following search string: (((hepatocellular carcinoma [MeSH Terms]) OR (hcc [MeSH Terms])) AND (microwave ablation)) AND (radiofrequency ablation). An updated literature search of conference proceedings of main international liver meetings was performed on 20 August 2020 to identify additional studies.

### 4.2. Data Abstraction and Quality Assessment

Data on study-, patient- and treatment-related characteristics were abstracted onto a standardized form, by two authors independently (AF, IC).

The risk of bias of individual studies was assessed independently by two authors (AF, IC) in the context of the primary outcome, based on the Cochrane tool for assessing the risk of bias of randomized trials [28]. Eventual disagreements were solved following a third opinion (RS).

### 4.3. Outcomes Assessed

The primary outcome was complete response, defined as complete ablation of the treated nodules (i.e., complete necrosis at the post-treatment imaging). Secondary outcomes were survival rate, defined as proportion of patients alive at different time-points (1, 2, 3, and 5 years), disease-free survival rate, defined as proportion of patients with no evidence of recurrence/progression at different time points (1, 2, 3, and 5 years), rate of local (i.e., within the same liver segment as the previously treated nodule) and distant (i.e., de novo lesions in other liver segments) recurrence, adverse event rate, and the number of treatment sessions.

### 4.4. Statistical Analysis

The two treatment groups were compared through a random-effects model based on the DerSimonian and Laird test [29], and results were expressed in terms of risk ratio (RR) in the case of dichotomous variables or mean difference in the case of continuous variables, along with the relevant 95% confidence intervals (CIs).

The presence of heterogeneity was measured in terms of I^2^ tests, with I^2^ < 20% interpreted as low-level heterogeneity and I^2^ between 20% and 50% interpreted as moderate heterogeneity. Any potential publication bias was verified through visual assessment of funnel plots.

Subgroup and sensitivity analyses in the context of the primary outcome were based on (a) mean max nodule size (≤3 cm versus >3 cm), (b) study location (East versus West or Africa), (c) restriction to high-quality trials.

In order to assess the impact of the mean max nodule size and the proportion of patients with a multifocal tumor on the primary outcome (complete response) and to explore eventual sources of heterogeneity, a meta-regression analysis based on the aforementioned variables was conducted.

All statistical analyses were conducted using RevMan version 5 from the Cochrane collaboration and R 3.0.2 (R Foundation for Statistical Computing, Vienna, Austria), and R 3.0.2 (R Foundation for Statistical Computing, Vienna, Austria), metafor package [30].

For all calculations, a two-tailed *p*-value of less than 0.05 was considered statistically significant.

## 5. Conclusions

The current meta-analysis shows that percutaneous MWA and RFA determine similar results in HCC patients. MWA seems to decrease the rate of long-term (5-year) recurrences, but this finding needs to be confirmed in further trials. If this finding is confirmed in future RCTs, MWA will likely play a role of paramount importance in the field of loco-regional treatments for HCC. However, in the absence of these data, a strong recommendation in favor of one treatment over the other cannot be given.

## Figures and Tables

**Table 1:** Characteristics of included randomized controlled trials comparing microwave ablation and radiofrequency ablation for the treatment of hepatocellular carcinoma.

Study, Year	Location; Time Period; Follow-Up	MWA, Number of Patients	RFA, Number of Patients	Multifocal/Bilobar Disease	Number of Nodules/Max Nodule Size (cm)
Abdelaziz, 2014 [18]	Egypt; NR; 2 years	AMICA^®^ GEM machine delivering frequency of 2450 MHz through 14 gages (150 and 200 mm) cooled shift electrodes; 66	Performed using 18 gauge (200 mm) internally Cool tip electrodes (Radionics^®^) connected to a 500-KHz radiofrequency generator; 45	MWA: 9 (13.6%)/0 (0%) RFA: 8 (17.7%)/2 (4.4%)	MWA: 76/2.9 ± 0.97 RFA: 52/2.95 ± 1.03
Chong, 2020 [19]	Hong Kong; 2011–2017; 5 years	Microwave needle with various power and duration setting depending on tumor size; 47	Cool-tip RFA needles of various sizes; 46	MWA: 4 (8.5%)/NR RFA: 7 (15.2%)/NR	MWA: NR/3.1 (2–4.5) RFA: NR/2.8 (2–5.5)
Kamal, 2019 [20]	Egypt; 2017–2018; 1 year	A 14 gauge 200 mm disposable MWA probe (AMICA probe MW) and a 2.45 GHz generator (AMICA^®^ GEN); 28	RITA StarBurst XL needle was used complying with manufacturer’s instructions; 28	MWA: 4 (14.2%)/NR RFA: 6 (21.4%)/NR	MWA: 34/3.25 ± 0.92 RFA: 34/3.28 ± 0.91
Qian 2012 [21]	China; 2009–2010; 1 year	MWA with 100 W for 8 min; 22	Single-application ablation in an automatic mode for 12 min; 20	MWA: 0 (0%)/0 (0%) RFA: 0 (0%)/0 (0%)	MWA: 22/2.1 ± 0.4 RFA: 20/2 ± 0.5
Shibata, 2002 [22]	Japan; 1999–2000; 2 years	MW generator which emits a 2450-MHz microwave, and a MW electrode 1.6 mm in diameter and 25 cm in length; 36	Monopolar-array 15-gauge needle electrode with 8 or 10 hook-shaped expandable electrode tines delivering 460-kHz frequency; 36	MWA: NR/NR RFA: NR/NR	MWA: 46/2.2 (0.9–3.4) RFA: 48/2.3 (1–3.7)
Vietti Violi, 2018 [23]	France/Switzerland; 2011–2015; 2 years	15-gauge liquid-cooled antenna and a 2·45 GHz generator with a power of 140 W; 71	200 W generator in the impedance control mode and a clustered internally cooled electrode; 73	MWA: 24 (33%)/NRRFA: 26 (35%)/NR	MWA: 98/1.8 ± 0.65 RFA: 104/1.8 ± 0.71
Yu, 2017 [24]	China; 2008–2015; 5 years	Cooled-shaft system; 203	Cooled-shaft system; 200	MWA: NR/NR RFA: NR/NR	MWA: 265/2.7 ± 1 RFA: 251/2.6 ± 1

Abbreviations: MWA—microwave ablation; NR—not reported; RFA—radiofrequency ablation.

**Table 2:** Subgroup and sensitivity analysis concerning the rate of complete ablation.

Variable	Subgroup	No. of Studies	No. of Patients	RR (95% CI)	Within-Group Heterogeneity (I^2^)
Nodule Size	≤3 cm	5	MWA: 402 RFA: 370	1.00 (0.99–1.01)	0%
	>3 cm	4	MWA: 105 RFA: 105	1.00 (0.87–1.13)	42%
Study Location	East	4	MWA: 380 RFA: 365	0.99 (0.95–1.03)	32%
	West/Africa	3	MWA: 208 RFA: 190	1.00 (0.96–1.05)	0%
Quality	High quality trials	3	MWA: 191 RFA: 198	0.98 (0.93–1.02)	0%

Abbreviations: CI, Confidence Interval; RR, Risk Ratio.

**Table 3:** Secondary outcomes.

Variable	Time Point	No. of Studies	No. of Patients	RR (95% CI)	Within-Group Heterogeneity (I^2^)
Survival Rate	1-year	4	MWA: 387 RFA: 364	1.05 (0.96–1.15)	81%
	2-year	3	MWA: 184 RFA: 164	1.03 (0.89–1.18)	14%
	3-year	2	MWA: 250 RFA: 246	0.99 (0.91–1.09)	0%
	5-year	2	MWA: 250 RFA: 246	0.91 (0.81–1.03)	0%
Disease-Free survival Rate	1-year	6	MWA: 407 RFA: 403	1.00 (0.96–1.04)	0%
	2-year	4	MWA: 220 RFA: 200	0.94 (0.84–1.06)	0%
	3-year	2	MWA: 250 RFA: 246	1.06 (0.93–1.21)	0%
	5-year	2	MWA: 250 RFA: 246	3.66 (1.32–42.27)	69%
Recurrence	Local Recurrence Rate	6	MWA: 426 RFA: 402	0.70 (0.43–1.14)	27%
	Distant Recurrence Rate	3	MWA: 116 RFA: 93	0.60 (0.39–0.92)	0%
Adverse Events	Adverse Event Rate	7	MWA: 473 RFA: 448	1.06 (0.48–2.34)	13%
Variable		No. of Studies	No. of patients	Mean Difference (95% CI)	Within-Group Heterogeneity (I^2^)
Number of Sessions		6	MWA: 407 RFA: 403	0.08 (−0.07 to 0.23)	92%

Abbreviation: CI, confidence interval; RR, risk ratio.

**Table 4:** Adverse events reported in the included trials.

Trial	MWA	RFA
Abdelaziz, 2014 [18]	Hematoma: 1 patient Burn: 1 patient	Hematoma: 2 patients Burn: 1 patient Pleural effusion: 2 patients
Chong, 2020 [19]	Ileus: 1 patient (2.1%)	Ascites: 1 patient (2.2%)
Kamal, 2019 [20]	Bleeding: 1 patient (3.6%) Hematemesis: 1 patient (3.6%)	None
Qian, 2012 [21]	None	None
Shibata, 2002 [22]	Abscesses: 2 patients Hematoma: 1 patient Cholangitis: 1 patient	Necrosis: 1 patient
Vietti Violi, 2018 [23]	Bleeding: 2 patients (2%)	Pneumothorax: 1 patient Bleeding: 1 patient Necrosis: 1 patient
Yu, 2017 [24]	Overall: 7 patients (3.4%)	Overall: 5 patients (2.5%)

Abbreviations: MWA—MicroWave Ablation; RFA—Radiofrequency Ablation.

## Supplementary Materials

The following are available online at https://www.mdpi.com/2072-6694/12/12/3796/s1, Figure S1: Risk of bias graph, Figure S2: Meta-regression plots, Table S1: Demographical and clinical characteristics of patients enrolled in the included randomized controlled trials.
