# Supplementary material for: Microwave Ablation Versus Radiofrequency Ablation for Treatment of Hepatocellular Carcinoma: A Meta-Analysis of Randomized Controlled Trials
Source: Cancers (Basel). 2020 Dec 16;12(12):3796. doi: 10.3390/cancers12123796 (PMC7766963; doi:10.3390/cancers12123796)
Supplement: Supplementary file 1 [file cancers-12-03796-s001.pdf]

Supplementary Materials: Microwave Ablation versus Radiofrequency Ablation for Treatment of Hepatocellular Carcinoma: A Meta-Analysis of Randomized Controlled Trials.

Antonio Facciorusso, Mohamed A. Abd El Aziz, Nicola Tartaglia, Daryl Ramai, Babu P Mohan, Christian Cotsoglou, Sara Pusceddu, Luca Giacomelli, Antonio Ambrosi and Rodolfo Sacco

Table S1. Demographical and clinical characteristics of patients enrolled in the included randomized controlled trials.

Study, Year	Age	Gender Male	Child-Pugh A/B/C	Performance Status 0	Alpha-fetoprotein ng/dL	Etiology Viral
Abdelaziz, 2014	MWA: 53.6 ± 5<br>RFA: 56.8 ± 7.2	MWA: 48 (72.7%)<br>RFA: 31 (68.9%)	MWA: 25 (37.9%)/41 (62.1%)<br>RFA: 24 (53.3%)/21 (46.7%)	MWA: 28 (42.4%)<br>RFA: 21 (46.7%)	MWA: 20 (5–3800)<br>RFA: 51 (5–12900)	NR
Chong, 2020	MWA: 63 (50–80)<br>RFA: 64.5 (42–85)	MWA: 30 (63.8%)<br>RFA: 38 (82.6%)	MWA: 39 (83%)/7 (14.9%)/ 1 (2.1%)<br>RFA: 40 (87%)/ 6 (13%)	NR	NR	MWA: 38 (80.9%)<br>RFA: 34 (73.9%)
Kamal, 2019	55 (42–80)	MWA: 75%<br>RFA: 78.6%)	MWA: 22 (78.6%)/6 (21.4%)<br>RFA: 22 (78.6%)/ 6 (21.4%)	MWA: 20 (71.4%)<br>RFA: 20 (71.4%)	MWA: 282.4 ± 469.7<br>RFA: 214.8 ± 319	NR
Qian, 2012	MWA: 52 ± 12<br>RFA: 56 ± 11	MWA: 20<br>RFA: 19	NR	NR	NR	NR
Shibata, 2002	MWA: 62.5 (52–74)<br>RFA: 63.6 (44–83)	MWA: 24<br>RFA: 26	MWA: 19/17<br>RFA: 21/15	NR	MWA: 4 >200<br>RFA: 7 >200	MWA: 36 (100%)<br>RFA: 36 (100%)

Vietti Violi, 2018	MWA: 68 (60–72)<br>RFA: 65 (59–73)	MWA: 59 (83%)<br>RFA: 62 (85%)	MWA: 57 (80%)/14 (20%)<br>RFA: 53 (73%)/ 20 (27%)	NR	MWA: 41 >10<br>RFA: 36 >10	MWA: 23 (32%)<br>RFA: 32 (43%)
Yu, 2017	NR	NR	NR	NR	NR	NR

Abbreviations: MWA—MicroWave Ablation; NR—Not Reported; RFA—Radiofrequency Ablation.

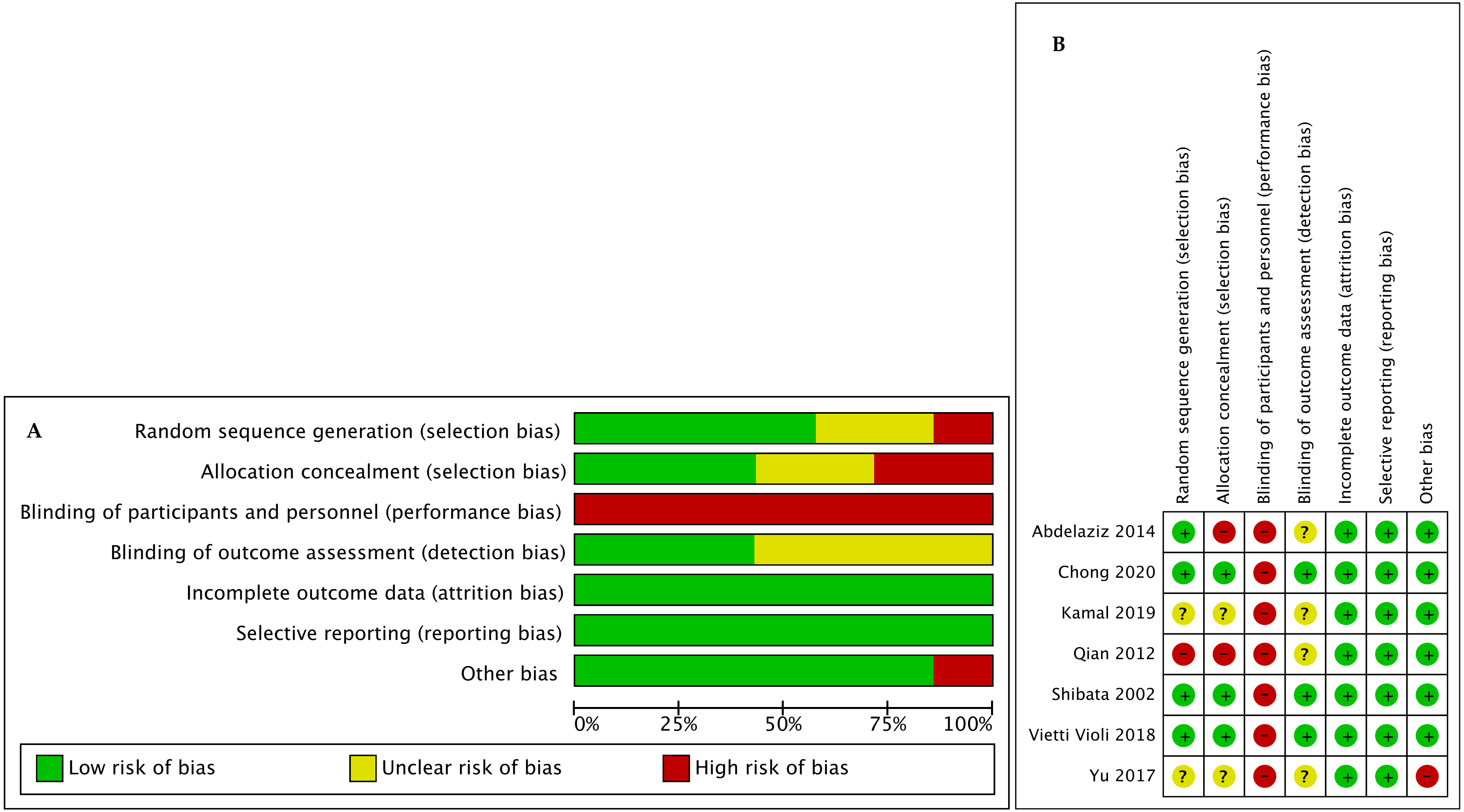

Figure S1. Risk of bias graph (1**A**) and summary (1**B**) in the included trials.

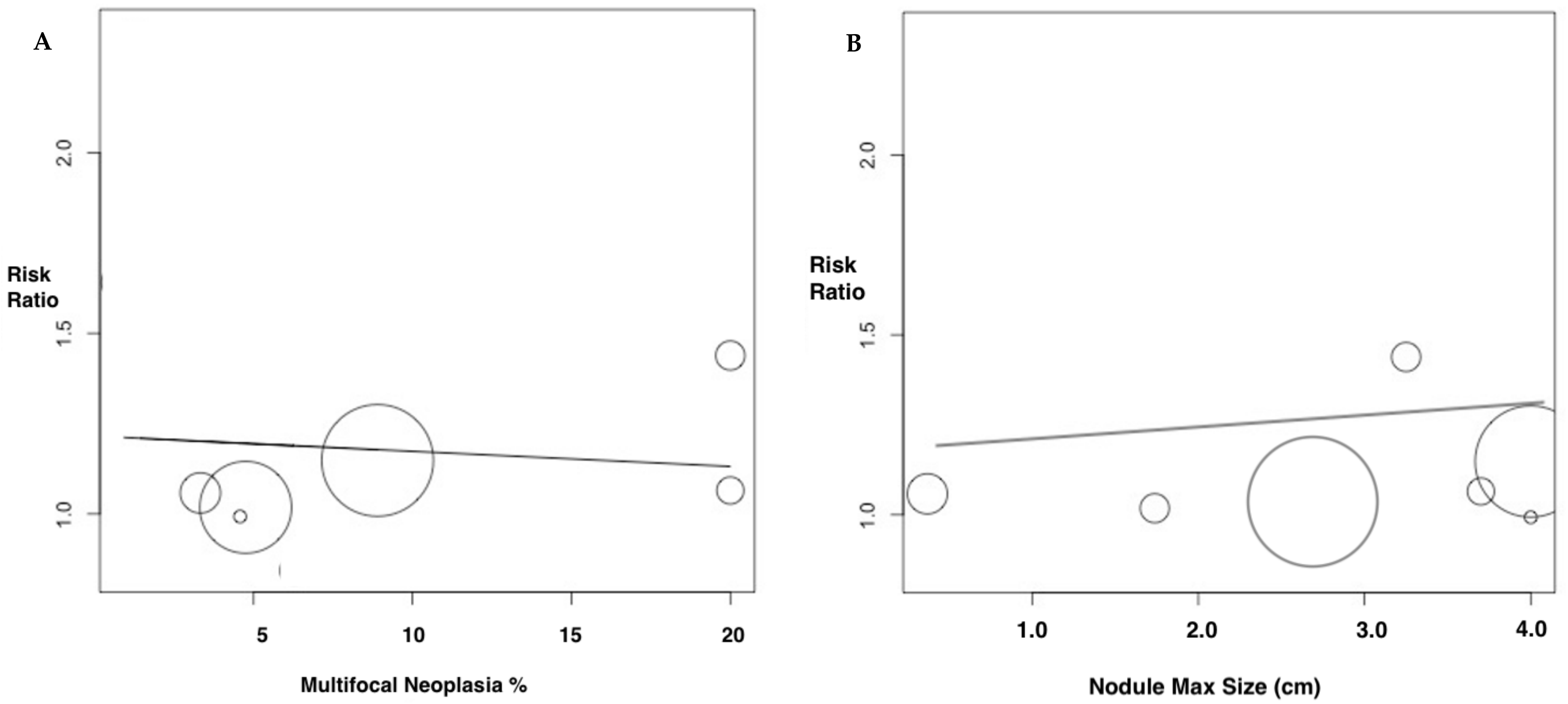

Figure S2. Meta-regression plots for the variable (**A**) proportion patients with multifocal neoplasia, (**B**) mean max nodule size.

Publisher's Note: MDPI stays neutral with regard to jurisdictional claims in published maps and institutional affiliations.

© 2020 by the authors. Licensee MDPI, Basel, Switzerland. This article is an open access article distributed under the terms and conditions of the Creative Commons Attribution (CC BY) license (http://creativecommons.org/licenses/by/4.0/).
